# Responsive Caregiving and Opportunities for Early Learning Associated With Infant Development: Results From a Prospective Birth Cohort in China

**DOI:** 10.3389/fped.2022.857107

**Published:** 2022-06-23

**Authors:** Ke Wang, Yue Qi, Qian Wei, Yuyang Shi, Yunhui Zhang, Huijing Shi

**Affiliations:** ^1^Key Laboratory of Public Health Safety, Ministry of Education, Department of Maternal, Child and Adolescent Health, School of Public Health, Fudan University, Shanghai, China; ^2^Key Laboratory of Public Health Safety, Ministry of Education, Department of Environmental Health, School of Public Health, Fudan University, Shanghai, China

**Keywords:** responsive caregiving, opportunities for early learning, infant development, suspected developmental delay, cohort study

## Abstract

**Background:**

Infant development shapes children’s health into adulthood. Although providing responsive caregiving and opportunities regarding early learning for infants have received increasing attention from the international community, few studies have been published on these topics thus far. The purpose of the present study, then, was to explore the influences of responsive caregiving and the opportunities for early learning on infant development.

**Methods:**

Mother-child dyads (3,714 pairs) were recruited from the Shanghai Maternal-Child Pairs Cohort (Shanghai MCPC) for the present study, and the development of infants, responsive caregiving and opportunities for early learning were collected from three waves of follow-up (2-, 6-, and 12-month old) We used the cross-lagged model to analyze the longitudinal correlation between responsive caregiving or opportunities for early learning and development of infants. We used the generalized estimation equation (GEE) to evaluate the effect of responsive caregiving and opportunities for early learning on suspected developmental delay; we also conducted a hierarchical analysis to investigate the interaction between responsive caregiving or opportunities for early learning and annual family income.

**Results:**

There was a mutual prediction between responsive caregiving or opportunities for early learning and some developmental domains of the Ages and Stages Questionnaires, third edition (ASQ-3). Sustained high-exposure to responsive caregiving or opportunities for early learning significantly decreased the risk of suspected developmental delay in most domains of the ASQ-3. And For infants whose annual family income was < ¥200,000, sustained high-exposure (Adjusted Odds Ratio = 0.456, 95% CI, 0.325–0.638) and fluctuating-exposure (Adjusted Odds Ratio = 0.510, 95% CI, 0.414–0.627) to responsive caregiving significantly reduced the risk for suspected developmental delay.

**Conclusion:**

Responsive caregiving or opportunities for early learning interacted with infant development. Infants’ early access to adequate responsive caregiving and opportunities for early learning exerted a sustained and positive impact on infant development, and this effect is more pronounced in relative low-income families.

## Introduction

Early childhood development refers to the continuous process in which children acquire cognition, language, movement, interpersonal communication, and emotional management from the embryonic period to 8 years of age (1, 2). A considerable number of children, however, cannot fully realize their early life developmental potential. The number of children suffering from insufficient early development in low- and middle-income countries (LMICs) in 2010 was estimated to be 250 million, accounting for 43% of all children until the age of five; of these, approximately 17 million were Chinese children (2). It is important to note that visual and auditory development peaks at ∼4 months; receptive and expressive languages peak at ∼9 months; more advanced cognitive development appears at ∼12 months; this is when the regular brain developmental processes of infants contribute to individual cognition, emotion, and social psychology in the later stages of life (3). Some studies have confirmed that the earlier the human capital investment in children occurs, the better the children can perform efficient learning tasks and productive activities (4, 5).

A majority of researchers have found that early childhood development is associated with some individual or environmental characteristics that entail genetic traits, maternal education and nutrition, infant and child nutrition, infectious diseases, environmental toxins, and disabilities; this also includes some psychosocial factors such as maternal depression, institutionalization, and exposure to societal violence (6–12). However, the Nurturing Care Framework for Early Childhood Development launched at the 71st World Health Assembly in 2018 introduced the concept of nurturing care, which comprised five components: good health, adequate nutrition, security and safety, responsive caregiving, and opportunities for early learning. There is increasing and compelling evidence for nurturing care as a critical opportunity to shape early childhood development (13). Responsive caregiving is defined as the caregiver’s appropriate feedback interaction when the child sends behavioral signals (14, 15). Opportunities for early learning refer to opportunities and various forms of stimulation for children’s early learning that are created by the family. The specific methods of responsive caregiving include encouraging a child’s behavior with positive emotions and oral statements and providing them with responsive and emotionally supportive interactions (13); these are affected by a satisfactory knowledge of child development and the emotional availability of caregivers (14, 16, 17).

In both high- and low-income countries, responsive caregiving was closely related to the improvement in children’s physical, cognitive, and psychosocial health; and has gradually become an important area of parenting (1, 18). One study reported that responsive caregiving can positively affect the healthy growth trajectory of infancy, thus potentially reducing the hazards associated with obesity (19–21). However, another analysis found no association between responsive caregiving and childhood linear growth (22). These authors suggested that responsive caregiving positively affected infant developmental outcomes such as cognition, language, and social-emotional traits (23). Also, more responsive caregiving was related to smoother social and emotional development at 2 years of age (22, 24), with a diminution in the number of behavioral problems at 3 years (25), higher intelligence at 4 and 12 years (26, 27), and improved academic performance at 7 years of age (18, 28). A study among Chilean school-age children found that strict parenting reduced children’s language proficiency test scores and increased children’s behavioral problems (29). However, most of the findings came from high-income countries, and few studies were conducted in LMICs.

There is a paucity of statistics on responsive caregiving and early learning opportunities for all of the countries and regions listed in the survey report released by the World Health Organization (WHO) in 2019. Moreover, studies designed to assess responsive caregiving and opportunities for early learning and impact on infant development are still lacking in China; whether any protective effect that is thereby produced is sustainable has not yet been clarified. Shanghai Maternal-Child Pairs Cohort (Shanghai MCPC), a prospective birth cohort established in April 2016 with focusing on encompassed the impacts of perinatal psychosocial stress, lifestyle, and nurturing family environment after delivery. Therefore, the principal objective of the present study was to explore the influences of responsive caregiving and opportunities for early learning on infant development.

## Materials and Methods

### Study Design and Participants

Data for present study came from ongoing Shanghai MCPC, which began in April 2016 with a context of the implementation of the comprehensive two-child policy after one-child policy for four decades in China (criteria and protocols for enrollment have been described before) (30). The original cohort that we focused on encompassed the impacts of perinatal psychosocial stress, lifestyle, and nurturing family environment after delivery; and on environmental-pollutant exposure on maternal and offspring health. A total of 6,714 pregnant women on whom pregnancy files were created for the first time at the maternity hospitals of Shanghai’s Pudong and Songjiang districts from April 2016 to June 2018 were continuously recruited into the cohort. Singleton live births (*n* = 5,481) were enrolled in the cohort after excluding withdrawal due to hospital transfer, physical conditions or other reasons, spontaneous abortion, therapeutically induced labor, or twin pregnancy; and were followed up at 2-, 6-, and 12-month old. In this study we collected data from three waves of follow-up measurements: by the end of October 2019, 4,230 follow-up questionnaires for 2-month-old infants; 3,470 follow-up questionnaires for 6-month infants; and 3,090 follow-up questionnaires for 12-month infants. Information on responsive caregiving, opportunities for early learning, and early childhood development was obtained through routine healthcare services using structured questionnaires, and questionnaires were filled out by the primary caregiver. Only the mother-child dyads who participated in the data collection for at least two of the waves were included. Ultimately, 3,714 pairs of mother-child dyads were included in the present study and Supplementary Figure 1 showed the details of the participants’ selection process. Completion of the questionnaires was voluntary, and all participants provided written informed consent. This study met the ethical guidelines of the two hospitals and was approved by the Ethics Committee of the School of Public Health, Fudan University.

### Measures

#### Responsive Caregiving

Based on the *Parenting guideline for children under three in Shanghai* (31) formulated by the Education Bureau of Shanghai Municipal People’s Government in accordance with the Nurturing Care Framework issued by WHO (13), we designed six items to evaluate the responsive caregiving infants received at 2 months old, including “create a clean and warm environment,” “keep the baby’s skin clean and dry,” “observe the baby’s eyes and navel,” “feed on demand and smile when feeding,” “give the baby touches and hugs,” and “communicate and play with the baby.” Each item used a four-level rating scale of 0–3 points (response options ranged from “never” to “always”), and a higher score indicated that the infant received greater responsive caregiving at 2 months of age. Cronbach’s alpha of all six items in this research sample was 0.863. The dimension of daily activities of Affordances in the Home Environment for Motor Development-Infant Scale (AHEMD-IS) was used to assess infants’ responsive caregiving at 6 and 12 months, and contained five items. A previous study using the Chinese version of AHEMD-IS showed that the validity of the scale was good (Cronbach α = 0.836) (32). For all questions, scores of 0 and 1 were awarded to the two response options of “no” and “yes,” and the total score ranged from 0 to 5 points. A higher score meant that the infant received greater responsive caregiving at 6 and 12 months of age (33, 34).

As there was no clear cut off point of levels of responsive caregiving up to now. Therefore, the present study set the cut off at 75th percentile of the cumulative scores according to the statistical consideration. The responsive caregiving groups of infants at 2, 6, and 12 months were divided as low-exposure (<P75) and high-exposure groups (≥P75). Furthermore, based on the 75th percentile of the total score in each wave of follow-up, infants were divided into a sustained high-exposure group (≥P75 in each wave), sustained low-exposure group (<P75 in each wave), and fluctuating-exposure group (≥P75 and <P75 across the three waves).

#### Opportunities for Early Learning

Using the *Parenting guideline for children under three in Shanghai* (31), we designed eight items to evaluate the opportunities for early learning of infants at two months of age, including “show the babies moving toys and faces,” “listen to soothing and gentle music,” “take the baby to participate in outdoor activities,” “provide colorful, non-toxic and hygienic toys,” “consciously allow the baby to practice the prone position, looking up”; and follow “eye-tracking,” “grasping,” and “rollover movements.” Each item used a four-level rating scale of 0–3 points (response options ranged from “never” to “always”), and a higher score indicated that the infant received greater opportunities for early learning at 2 months. Cronbach’s alpha for all eight items in this research sample was 0.897. The dimensions of Variety of Stimulation, Fine-Motor Toys, and Gross-Motor Toys in AHEMD-IS were used to assess opportunities for early learning of infants at 6 and 12 months old. The six items included in the dimension of Variety of Stimulation were graded. For the first four items, scores of 3, 2, 1, and 0 points were awarded to the four response options of “never,” “sometimes,” “often,” and “always.” The remaining two items were scored in reverse, with a total score of 0–18 points. A higher score indicated that the families did not restrict their babies’ activities much, and the children would be more encouraged to move freely and to explore actively. There were 11 items included in the dimension of Fine-Motor Toys and Gross-Motor Toys in AHEMD-IS, and each item was scored on a scale of 0–3 points. The scores from low to high corresponded to the options “none,” “1–2,” “3–4,” and “5 or more,” respectively. A higher score signified more game materials for the infant. The total score for Variety of Stimulation, Fine-Motor Toys, and Gross-Motor Toys reflected the opportunities for early learning of infants at 6 and 12 months of age, and a higher score meant richer opportunities for the early learning of infants (33, 34).

The grouping of opportunities for early learning was consistent with the grouping rules of the responsive caregiving groups.

#### Infant Development

The development of infants 2, 6, and 12 months old was evaluated using the Ages and Stages Questionnaires, third edition (ASQ-3), which includes five developmental domains: communication, gross motor, fine motor, problem-solving, and personal-social. Each domain contains six items, and for each item, scores of 10, 5, and 0 points were awarded to the three response options of “yes,” “sometimes,” and “not yet.” Suspected developmental delay in each domain was indicated by domain total scores ≤ 2 SDs below the mean, and if the number of domains of suspected developmental delay was > 1, it was defined as suspected infant developmental delay (35). A previous study on the reliability and validity of the Chinese version of the ASQ-3 in a nationally representative normative sample showed that the Chinese version of the ASQ-3 is a reliable and valid measure with a representative national sample aged 1–66 months, and can be used for to screen and monitor the development of children in the mainland of China (Cronbach α = 0.8) (36).

### Control Variables

A self-administered questionnaire was used to collect maternal age, educational level, and annual family income at 12–16 weeks of pregnancy. Gestational week, delivery mode, infant sex, and birthweight were obtained through the obstetrical records of the hospital. The gestational week of delivery was obtained by calculating the time difference between the first day of the last menses and the birth date. A gestational week of delivery less than 37 weeks was termed preterm delivery; and there were two methods of delivery, natural and cesarean section. The infant’s birthweight was measured and recorded by the obstetrical nurse according to the standard procedure within 1 h after birth, with an accuracy of 10 g and a normal birthweight range of 2,500–4,000 g.

### Statistical Analyses

We implemented descriptive statistics, normality testing, and used the generalized estimation equation (GEE) with IBM SPSS Statistics (version 21); and the cross-lagged model was performed with Mplus (version 7.0). Missing covariate data were imputed using multiple imputation. The statistical description of continuous variables following normal distribution were means and standard deviation (SD); while the statistical description of continuous variables in a non-normal distribution was P_50_ (P_25_, P_75_), and our classification variables were designated as frequencies with percentages. After controlling for covariates, the cross-lagged model was exploited to explore the longitudinal associations between responsive caregiving or opportunities for early learning and development of infants at 2, 6, and 12 months of age. The GEE was used to evaluate the effects of responsive caregiving and opportunities for early learning on suspected developmental delay in five domains of the ASQ-3, and a hierarchical analysis was conducted according to annual family income to investigate the interaction between responsive caregiving and opportunities for early learning and annual family income.

## Results

### Sample Characteristics

**FIGURE 1:**
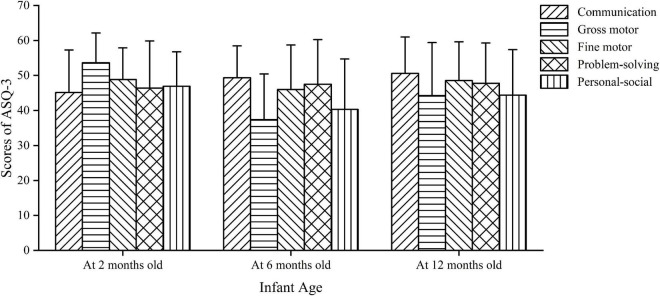
Distribution of scores of developmental domains of ASQ-3 for infants at 2, 6, and 12 months of age.

The demographic characteristics of samples and repeated responsive caregiving and opportunities for early learning of participants across the three waves are presented in Supplementary Table 1. The scores in communication of infants at 2 months (45.15 ± 12.12), 6 months (49.35 ± 9.13), and 12 months of age (50.60 ± 10.39) gradually increased, and the same trend was seen in problem-solving. The scores in gross motor of infants at 2, 6, and 12 months were 53.65 ± 8.51, 37.38 ± 13.05, and 44.27 ± 15.13, respectively. The scores in fine motor of infants at three waves were 48.87 ± 9.02, 46.00 ± 12.73, and 48.56 ± 11.09, respectively. The scores in personal-social of infants at three waves were 46.93 ± 9.84, 40.32 ± 14.41, and 44.38 ± 13.01, respectively (Figure 1). The rates of suspected developmental delay in a single domain ranged from 2.8 to 6.6%; and the overall rates of suspected developmental delay at 2, 6, and 12 months were 15.6, 15.8, and 12.6%, respectively (Table 1).

**TABLE 1:** Suspected developmental delay in each domain of ASQ-3 and suspected infant developmental delay at 2, 6, and 12 months of age.

Variable	At 2 months old (*n* = 3,445)	At 6 months old (*n* = 3,199)	At 12 months old (*n* = 2,922)
Suspected developmental delay in communication	121 (3.5)	195 (6.1)	85 (2.9)
Suspected developmental delay in gross motor	227 (6.6)	112 (3.5)	137 (4.7)
Suspected developmental delay in fine motor	193 (5.6)	138 (4.3)	143 (4.9)
Suspected developmental delay in problem-solving	117 (3.4)	157 (4.9)	85 (2.9)
Suspected developmental delay in personal-social	96 (2.8)	125 (3.9)	94 (3.2)
Suspected infant developmental delay	537 (15.6)	505 (15.8)	368 (12.6)

### Responsive Caregiving, Opportunities for Early Learning, and Infant Development

**FIGURE 2:**
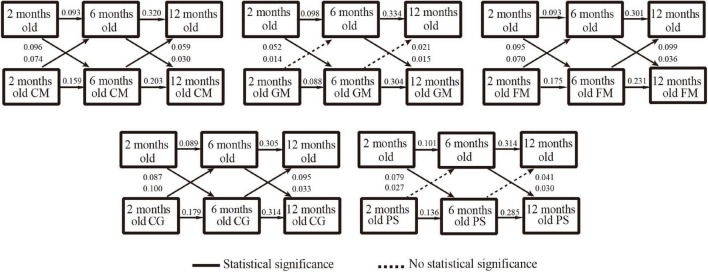
Results of cross-lag analysis between responsive caregiving and development of infants. CM, communication; GM, gross motor; FM, fine motor; CG, problem-solving; PS, personal-social.

**FIGURE 3:**
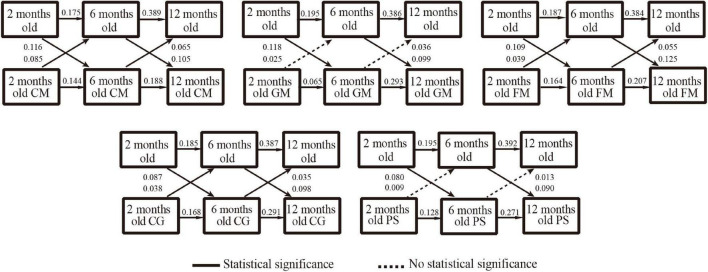
Results of cross-lag analysis between opportunities for early learning and development of infants. CM, communication; GM, gross motor; FM, fine motor; CG, problem-solving; PS, personal-social.

As detailed in Figures 2, 3, after controlling for maternal age, maternal educational level, annual family income, infant sex, delivery mode, gestational age, and birthweight, the cross-lagged models indicated significant and positive cross-lags linking responsive caregiving or opportunities for early learning exposure at 2 months old, with higher scores in communication (β = 0.096, *P* < 0.001; β = 0.116, *P* < 0.001), gross-motor (β = 0.052, *P* = 0.033; β = 0.118, *P* < 0.001), fine-motor (β = 0.095, *P* < 0.001; β = 0.109, *P* < 0.001), problem-solving (β = 0.087, *P* < 0.001; β = 0.087, *P* < 0.001), and personal-social (β = 0.079, *P* = 0.001; β = 0.080, *P* < 0.001) domains at 6 months. Similarly, responsive caregiving or opportunities for early learning exposure at 6 months was associated with higher scores in communication (β = 0.030, *P* < 0.001; β = 0.105, *P* < 0.001), gross motor (β = 0.015, *P* = 0.034; β = 0.099, *P* < 0.001), fine motor (β = 0.036, *P* < 0.001; β = 0.125, *P* < 0.001), problem-solving (β = 0.033, *P* < 0.001; β = 0.098, *P* < 0.001), and personal-social (β = 0.030, *P* = 0.002; β = 0.090, *P* < 0.001) domains at 12 months. Infants who exhibited better performance in communication, fine-motor, or problem-solving domains scored higher points for responsive caregiving and opportunities for early learning across all three waves.

### Responsive Caregiving, Opportunities for Early Learning, and Suspected Infant Developmental Delay

Table 2 depicts the GEE regression results with an independent working correlation matrix. After controlling for maternal age at delivery, maternal educational level, annual family income, delivery mode, premature delivery, infant sex, and birthweight, we found that compared to sustained low-exposure to the responsive caregiving group, the sustained high-exposure group carried a significantly diminished risk of developmental delay in the five domains of the ASQ-3 and the same as for the fluctuating-exposure group. However, sustained high-exposure to opportunities in the early learning group exhibited a lower risk of developmental delay than the sustained low-exposure group in most domains of the ASQ-3 except for communication, and the fluctuating-exposure group only showed a significantly decreased risk of developmental delay in the fine motor and personal-social domains compared with the sustained low-exposure group.

**TABLE 2:** The relations between responsive caregiving or opportunities for early learning and infant development delay.

Variable	Communication	Gross motor	Fine motor	Problem-solving	Personal-social
					
	aOR^†^ (95%CI)	aOR^†^ (95%CI)	aOR^†^ (95%CI)	aOR^†^ (95%CI)	aOR^†^ (95%CI)
Responsive caregiving (1 vs. 3)	0.638 (0.476∼0.856)**	0.612 (0.468∼0.803)***	0.583 (0.441∼0.771)***	0.412 (0.285∼0.598)***	0.643 (0.455∼0.910)*
Responsive caregiving (2 vs. 3)	0.554 (0.337∼0.913)*	0.571 (0.361∼0.905)*	0.379 (0.228∼0.629)***	0.270 (0.133∼0.549)***	0.361 (0.181∼0.717)**
Opportunities for early learning (1 vs. 3)	0.767 (0.547∼1.077)	0.807 (0.600∼1.085)	0.717 (0.523∼0.983)*	0.736 (0.511∼1.059)	0.668 (0.451∼0.989)*
Opportunities for early learning (2 vs. 3)	0.502 (0.249∼1.013)	0.425 (0.209∼0.865)*	0.270 (0.112∼0.652)**	0.235 (0.077∼0.720)*	0.230 (0.073∼0.727)*

^†^Adjustment for maternal age at delivery, maternal education level, annual family income, the infant sex, preterm, delivery mode, and birthweight. Responsive caregiving (1 to fluctuating-exposure, 2 to sustained high-exposure, 3 to sustained low-exposure), opportunities for early learning (1 to fluctuating-exposure, 2 to sustained high-exposure, 3 to sustained low-exposure). *P < 0.05, **P < 0.01, ***P < 0.001.

For infants whose annual family income was < ¥200,000, sustained high exposure (aOR = 0.456, 95% CI, 0.325–0.638) and fluctuating exposure (aOR = 0.510, 95% CI, 0.414–0.627) to responsive caregiving significantly reduced the risk of suspected infant developmental delay; yet, the effect was not significant in the group with an annual family income ≥ ¥200,000. We uncovered significant interaction between the exposure level of opportunities for early learning and the annual family income on the early development of infants, regardless of annual family income. For infants whose annual family income was < ¥200,000, sustained high-exposure (aOR = 0.298, 95% CI, 0.176–0.504) and fluctuating-exposure (aOR = 0.742, 95% CI, 0.585–0.942) to opportunities for early learning significantly reduced the risk of suspected infant developmental delay. For infants whose annual family income was ≥ ¥200,000, sustained high-exposure (aOR = 0.373, 95% CI, 0.180–0.773) and fluctuating-exposure (aOR = 0.681, 95% CI, 0.473–0.981) to opportunities for early learning significantly reduced the risk of suspected infant developmental delay (Table 3).

**TABLE 3:** The relations between responsive caregiving or opportunities for early learning and development of infants in different income groups.

Variable	Annual family income < ¥200,000	Annual family income ≥ ¥200,000
	aOR^†^ (95%CI)	aOR^†^ (95%CI)
Responsive caregiving (1 vs. 3)	0.510 (0.414∼0.627)***	1.129 (0.815∼1.565)
Responsive caregiving (2 vs. 3)	0.456 (0.325∼0.638)***	0.700 (0.402∼1.220)
Opportunities for early learning (1 vs. 3)	0.742 (0.585∼0.942)*	0.681 (0.473∼0.981)*
Opportunities for early learning (2 vs. 3)	0.298 (0.176∼0.504)***	0.373 (0.180∼0.773)**

^†^Adjustment for maternal age at delivery, maternal education level, the infant sex, preterm, delivery mode, and birthweight. Responsive caregiving (1 to fluctuating-exposure, 2 to sustained high-exposure, 3 to sustained low-exposure), opportunities for early learning (1 to fluctuating-exposure, 2 to sustained high-exposure, 3 to sustained low-exposure). *P < 0.05, **P < 0.01, ***P < 0.00.

## Discussion

Many longitudinal studies have confirmed the impact of family rearing on children’s development. For example, one study reported that parent-child interaction and books exerted positive impacts on the development of children in poor rural areas of China (37), and another study among Chinese children found that parental rearing efficacy was related to children’s social development (38). Furthermore, a recent study revealed that maternal sensitivity at 10 months was beneficial to the development of hot and cold executive function at 48 months of age (39). Another study that was similar in design to the present study and also entailed a cross-lagged model to analyze the relationship between ASQ development and responsive caregiving in Korean children at 2 years of age, found that responsive caregiving exerted a sustained positive effect on the development of communication regions in infants and young children (40). A small number of studies showed the impact of child development on family rearing. For example, a Japanese study confirmed that a child’s problem behavior at 7.5 years old predicted the caregiver’s overreacting parenting style at 9 years (41). Investigators in a study in South Korea ascertained that the development domain of problem-solving in infants at 4 months positively predicted responsive caregiving at 13 months (40). Only a few groups found that there was an interaction between family rearing and child development. Melissa et al. found that sensitive maternal parenting at 24 months promoted language development at 36 months; however, receptive language development at the age of 24 months affected the mother’s sensitive parenting behavior at the age of 36 months only in the case of male children (42). A follow-up study of Turkish children established a negative correlation between mother’s responsive caregiving for children aged three and aggressive behavior within 5 years; the aggressive behavior of children at the age of 5 and 6 predicted the level of responsive caregiving in the subsequent period (43). In this study, we advanced the science in this area by estimating repeated outcomes that included early developmental level, responsive caregiving, and opportunities for early learning of infants at 2, 6, and 12 months. Furthermore, we demonstrated that responsive caregiving or opportunities for early learning at time T positively predicted the scores of the five developmental domains of ASQ-3 at time T + 1. In addition, the scores of corresponding developmental domains of the ASQ-3 at time T also positively predicted the score of responsive caregiving or opportunities for early learning at time T + 1; i.e., there was a mutual prediction between the two variables. The findings of this study provided evidence toward the theory of continuous interaction between family rearing and child development, although additional in-depth research is needed in the future to reveal their relationship.

Another finding of our study was that adequate and sustained responsive caregiving was beneficial to infants with relatively lower family income, and that this can significantly reduce the risk of suspected growth retardation; however, this effect was not noted in infants with relatively higher family income. Rich opportunities for early learning also significantly improve the level of the early development of infants with different family incomes, suggesting that it is necessary to provide appropriate opportunities for early learning for infants in the family environment, regardless of family income; additionally, the provision of a high level of responsive caregiving is particularly important in promoting the early development of infants with relatively lower family income. Family-centered child-rearing and caring emphasize that parents play an irreplaceable role in the early development of children. Parents’ participation not only reduces the high cost of preschool education but also establishes a parent-child relationship that can benefit children for life. Some studies conducted in families with lower socioeconomic status showed similar findings. For example, a follow-up study conducted in the United States found that high-quality family rearing significantly improved the cognitive development of children in vulnerable groups (44). A randomized controlled trial revealed that providing family parenting support to low-income groups in New York City reduced the incidence of children’s problem behaviors and interrupted the vicious circle of poverty (45). In a review of the results of 23 parenting intervention projects aimed at promoting child health and equity in Europe, the authors demonstrated an improvement in children’s health, fine motor development, and cognitive function (46). In summary, to overcome the inequity of children’s health caused by differences in social and economic status, it is necessary to carry out scientific child-rearing knowledge and skills training for child caregivers of urban families in China. Particular attention should also be paid to improving the responsive caregiving of families with relatively lower income, to reduce the damage to children’s health caused by low socio-economic status-thereby promoting the realization of infant health equity.

### Strengths and Limitations

This study possessed several strengths. First, we used a prospective birth-cohort study with a relatively large sample size and a superior causally demonstrative ability relative to other observational and epidemiologic studies. Moreover, this was an early longitudinal study conducted in urban areas of China to explore the effects of responsive caregiving and opportunities for early learning on infant development; we repeatedly measured early developmental levels, responsive caregiving, and opportunities for early learning of infants at 2, 6, and 12 months of age. Additionally, using the GEE and cross-lag model we found that responsive caregiving and opportunities for early learning exerted a continuous and positive impact on infant development. This study also bore the following shortcomings. First, the scope of this study was limited to the two districts of Shanghai, and the subjects of this study were mainly from the suburbs of Shanghai. Therefore, the generalization of the conclusion is limited. In the future, investigators should increase their numbers of research subjects from different regions of eastern, central, and western China to facilitate the proliferation of data throughout the country. Second, our use of an inconsistent assessment tool for responsive caregiving and opportunities for early learning at 2, 6, and 12 months was another limitation. In the next phase of our study, we plan to develop a series of evaluation tools with reliability and validity in accordance with the social backgrounds of our country and apply them comprehensively. Third, according to the survey results of “Shanghai Yearbook 2019,” the per capita disposable income of urban households is ¥68,034. Considering that each household has at least 3 people in this study, Annual household income was divided into two categories: below ¥200,000 and above ¥200,000. In this process of converting continuous variables into dichotomous variables, some information would be lost. Meanwhile, we adjusted for several potential confounders, such as socio-demographic data, maternity information, socioeconomic parameters to improve the reliability of the results, however, we regret that no information was collected on children’s own energy intake, which is recognized as an important factor in children’s development. In addition, the assessment of responsive parenting and child development in this study used questionnaires only and no laboratory data were obtained. Further studies are therefore needed to observe the long-term effects of responsive caregiving and opportunities for early learning on child development. Finally, the present study only examined the relationship between two aspects of the nurturing care and infant development. Further studies are required to use dimensional analysis to investigate the relationship between all aspects of the nurturing care and child development.

## Conclusion

In conclusion, Through the examination of the influences of responsive caregiving and opportunities for early learning on infant development, we demonstrated that infants’ early access to adequate responsive caregiving and opportunities for early learning impacted infant development in sustained and positive fashions. Further, we noted continuous interactions between responsive caregiving or opportunities for early learning and infants’ developmental domains of communication, fine-motor, and problem-solving. Furthermore, sustained high-exposure to responsive caregiving within 1 year after birth significantly reduced the risk of suspected developmental delay in infants with relatively lower household income. As such, regardless of family income, sustained high-exposure to opportunities for early learning was beneficial in curtailing the risk of suspected developmental delay in infants.

## Data Availability Statement

The data analyzed in this study is subject to the following licenses/restrictions: The datasets presented in this article will be available for investigators after approval by Fudan University. Requests to access these datasets should be directed to the corresponding author.

## Ethics Statement

The study was conducted according to the guidelines of the Declaration of Helsinki, and approved by the Institutional Review Board in Public Health School of Fudan University in April 2016 and March 2020, respectively (IRB numbers 2016-04-0587 and 2016-04-0587-EX). Written informed consent to participate in this study was provided by the participants’ legal guardian/next of kin.

## Author Contributions

HS and YZ conducted project administration. HS, KW, and YQ designed the study. KW and YQ conducted the data analysis and interpretation. KW, QW, and YS drafted the manuscript for publication with the assistance of HS oversaw the original data collection. All authors have read and approved the final manuscript and critically reviewed and approved the final manuscript.

## Conflict of Interest

The authors declare that the research was conducted in the absence of any commercial or financial relationships that could be construed as a potential conflict of interest.

## Publisher’s Note

All claims expressed in this article are solely those of the authors and do not necessarily represent those of their affiliated organizations, or those of the publisher, the editors and the reviewers. Any product that may be evaluated in this article, or claim that may be made by its manufacturer, is not guaranteed or endorsed by the publisher.

## References

[1] Black MM Walker SP Fernald LCH Andersen CT DiGirolamo AM Lu CL Early childhood development coming of age: science through the life course. *Lancet.* (2017) 389:77–90. 10.1016/S0140-6736(16)31389-7 27717614 PMC5884058

[2] Lu CL Black MM Richter LM. Risk of poor development in young children in low-income and middle-income countries: an estimation and analysis at the global, regional, and country level. *Lancet Glob Health.* (2016) 4:E916–22. 10.1016/S2214-109X(16)30266-2 27717632 PMC5881401

[3] Thompson RA Nelson CA. Developmental science and the media - Early brain development. *Am Psychol.* (2001) 56:5–15. 11242988 10.1037/0003-066x.56.1.5

[4] Richter LM Daelmans B Lombardi J Heymann J Boo FL Behrman JR Investing in the foundation of sustainable development: pathways to scale up for early childhood development. *Lancet.* (2017) 389:103–18. 10.1016/S0140-6736(16)31698-1 27717610 PMC5880532

[5] Bakermans-Kranenburg MJ van Ijzendoorn MH Juffer F. Earlier is better: a meta-analysis of 70 years of intervention improving cognitive development in institutionalized children. *Monogr Soc Res Child Dev.* (2008) 73:279–93. 10.1111/j.1540-5834.2008.00498.x 19121021

[6] Walker SP Wachs TD Grantham-McGregor S Black MM Nelson CA Huffman SL Child development 1 inequality in early childhood: risk and protective factors for early child development. *Lancet.* (2011) 378:1325–38. 10.1016/S0140-6736(11)60555-2 21944375

[7] Chamberlain C O’Mara-Eves A Porter J Coleman T Perlen SM Thomas J Psychosocial interventions for supporting women to stop smoking in pregnancy. *Cochr Database Syst Rev.* (2017) 2:CD001055. 10.1002/14651858.CD001055.pub5 28196405 PMC6472671

[8] Imdad A Yakoob MY Bhutta ZA. The effect of folic acid, protein energy and multiple micronutrient supplements in pregnancy on stillbirths. *BMC Public Health.* (2011) 11:S4. 10.1186/1471-2458-11-S3-S4 21501455 PMC3231910

[9] Kvalevaag AL Ramchandani PG Hove O Assmus J Eberhard-Gran M Biringer E. Paternal mental health and socioemotional and behavioral development in their children. *Pediatrics.* (2013) 131:E463–9. 10.1542/peds.2012-0804 23296445

[10] Stein A Pearson RM Goodman SH Rapa E Rahman A McCallum M Effects of perinatal mental disorders on the fetus and child. *Lancet.* (2014) 384:1800–19. 10.1016/S0140-6736(14)61277-0 25455250

[11] Horta BL de Mola CL Victora CG. Breastfeeding and intelligence: a systematic review and meta-analysis. *Acta Paediatr.* (2015) 104:14–9. 10.1111/apa.13139 26211556

[12] Victora CG Horta BL de Mola CL Quevedo L Pinheiro RT Gigante DP Association between breastfeeding and intelligence, educational attainment, and income at 30 years of age: a prospective birth cohort study from Brazil. *Lancet Glob Health.* (2015) 3:E199–205. 10.1016/S2214-109X(15)70002-1 25794674 PMC4365917

[13] Britto PR Lye SJ Proulx K Yousafzai AK Matthews SG Vaivada T Nurturing care: promoting early childhood development. *Lancet.* (2017) 389:91–102. 10.1016/S0140-6736(16)31390-3 27717615

[14] Rasheed MA Yousafzai AK. The development and reliability of an observational tool for assessing mother-child interactions in field studies- experience from Pakistan. *Child Care Health Dev.* (2015) 41:1161–71. 10.1111/cch.12287 26350208

[15] Landry SH Smith KE Swank PR Zucker T Crawford AD Solari EF. The effects of a responsive parenting intervention on parent-child interactions during shared book reading. *Dev Psychol.* (2012) 48:969–86. 10.1037/a0026400 22122475

[16] Browne JV Talmi A. Family-based intervention to enhance infant-parent relationships in the neonatal intensive care unit. *J Pediatr Psychol.* (2005) 30:667–77. 10.1093/jpepsy/jsi053 16260436

[17] Tripathy P Nair N Barnett S Mahapatra R Borghi J Rath S Effect of a participatory intervention with women’s groups on birth outcomes and maternal depression in Jharkhand and Orissa, India: a cluster-randomised controlled trial. *Lancet.* (2010) 375:1182–92. 10.1016/S0140-6736(09)62042-0 20207411

[18] Eshel N Daelmans B de Mello MC Martines J. Responsive parenting: interventions and outcomes. *Bull World Health Organ.* (2006) 84:991–8. 10.2471/blt.06.030163 17242836 PMC2627571

[19] Paul IM Savage JS Anzman-Frasca S Marini ME Beiler JS Hess LB Effect of a responsive parenting educational intervention on childhood weight outcomes at 3 years of age the insight randomized clinical trial. *JAMA.* (2018) 320:461–8. 10.1001/jama.2018.9432 30088009 PMC6142990

[20] Savage JS Hohman EE Marini ME Shelly A Paul IM Birch LL. INSIGHT responsive parenting intervention and infant feeding practices: randomized clinical trial. *Int Behav Nutr Phys Act.* (2018) 15:64. 10.1186/s12966-018-0700-6 29986721 PMC6038199

[21] Marshall Z Delahunty C. The insight responsive parenting intervention reduced infant weight gain and overweight status. *Arch Dis Child Educ Pract Ed.* (2018) 103:57–8. 10.1136/archdischild-2017-313112 28684547

[22] Scherer E Hagaman A Chung E Rahman A O’Donnell K Maselko J. The relationship between responsive caregiving and child outcomes: evidence from direct observations of mother-child dyads in Pakistan. *BMC Public Health.* (2019) 19:252. 10.1186/s12889-019-6571-1 30819173 PMC6396475

[23] Yousafzai AK Rasheed MA Rizvi A Armstrong R Bhutta ZA. Effect of integrated responsive stimulation and nutrition interventions in the lady health worker programme in Pakistan on child development, growth, and health outcomes: a cluster-randomised factorial effectiveness trial. *Lancet.* (2014) 384:1282–93. 10.1016/S0140-6736(14)60455-4 24947106

[24] Yousafzai AK Obradovic J Rasheed MA Rizvi A Portilla XA Tirado-Strayer N Effects of responsive stimulation and nutrition interventions on children’s development and growth at age 4 years in a disadvantaged population in Pakistan: a longitudinal follow-up of a cluster-randomised factorial effectiveness trial. *Lancet Glob Health.* (2016) 4:E548–58. 10.1016/S2214-109X(16)30100-0 27342433

[25] Bakeman R Brown JV. Early interaction - consequences for social and mental-development at 3 years. *Child Dev.* (1980) 51:437–47. 10.2307/1129277 7398451

[26] Beckwith L Rodning C Cohen S. Preterm children at early adolescence and continuity and discontinuity in maternal responsiveness from infancy. *Child Dev.* (1992) 63:1198–208. 10.1111/j.1467-8624.1992.tb01689.x 1446549

[27] Landry SH Smith KE Swank PR Assel MA Vellet S. Does early responsive parenting have a special importance for children’s development or is consistency across early childhood necessary? *Dev Psychol.* (2001) 37:387–403. 10.1037//0012-1649.37.3.387 11370914

[28] Bradley RH. HOME measurement of maternal responsiveness. *New Dir Child Dev.* (1989) 43:63–73. 10.1002/cd.23219894307 2523523

[29] Berthelon M Contreras D Kruger D Palma MI. Harsh parenting during early childhood and child development. *Econ Hum Biol.* (2020) 36:100831. 10.1016/j.ehb.2019.100831 31816562

[30] Yingya Z Yuhan Z Qingyang Z Bing X Wenjuan M Xirong X Determination of antibiotic concentration in meconium and its association with fetal growth and development. *Environ Int.* (2019) 123:70–8. 10.1016/j.envint.2018.11.053 30500730

[31] Shanghai Municipal Education Commission Office. *0^~^3 Years Old Infant Education Program in Shanghai.* (2008). Available online at: http://edu.sh.gov.cn/web/xxgk/rows_content_view.html?article_code=402022008002 (accessed January 15, 2022).

[32] Jing H Jie M Wei C Zhuochun W. Reliability and validity of affordances in the home environment for motor development-infant scale-Chinese version. *Chin J Child Health Care.* (2018) 10:1064–7. 10.11852/zgetbjzz2018-26-10-06

[33] Cacola PM Gabbard C Montebelo MIL Santos DCC. The new affordances in the home environment for motor development - infant scale (AHEMD-IS): versions in English and Portuguese languages. *Braz Phys Ther.* (2015) 19:507–25. 10.1590/bjpt-rbf.2014.0112 26647753 PMC4668345

[34] Cacola PM Gabbard C Montebelo MIL Santos DCC. Further development and validation of the affordances in the home environment for motor development-infant scale (AHEMD-IS). *Phys Ther.* (2015) 95:901–23. 10.2522/ptj.20140011 25524875

[35] Agarwal PK Xie HC Rema ASS Rajadurai VS Lim SB Meaney M Evaluation of the ages and stages questionnaire (ASQ 3) as a developmental screener at 9, 18, and 24 months. *Early Hum Dev.* (2020) 147:105081. 10.1016/j.earlhumdev.2020.105081 32502946

[36] Mei W Xiaoyan B Squires J Guoying Y Xiaochuan W Huichao X Studies of the norm and psychometrical properties of the ages and stages questionnaires, third edition, with a Chinese national sample. *Chin. J. Pediatr.* (2015) 12:913–8. 10.3760/cma.j.issn.0578-1310.2015.12.009 26887546

[37] Zhang JX Guo SF Li Y Wei QW Zhang CH Wang XL Factors influencing developmental delay among young children in poor rural China: a latent variable approach. *BMJ Open.* (2018) 8:e021628. 10.1136/bmjopen-2018-021628 30173158 PMC6120651

[38] Yang SM Yue H Nie LL Wu RY Liu Y Yan C Association between parental self-efficacy consistency and social development of children. *Chin J Sch Health.* (2021) 42:1660–4. 10.16835/j.cnki.1000-9817.2021.11.014

[39] Frick MA Forslund T Brocki KC. Does child verbal ability mediate the relationship between maternal sensitivity and later self-regulation? A longitudinal study from infancy to 4 years. *Scand J Psychol.* (2019) 60:97–105. 10.1111/sjop.12512 30625240

[40] Cha K. Relationships among negative emotionality, responsive parenting and early socio-cognitive development in Korean children. *Infant Child Dev.* (2017) 26:e1990. 10.1002/icd.1990

[41] Suzuki K Kita Y Kaga M Takehara K Misago C Inagaki M. The association between children’s behavior and parenting of caregivers: a longitudinal study in Japan. *Front Public Health.* (2016) 4:17. 10.3389/fpubh.2016.00017 26913279 PMC4753532

[42] Barnett MA Gustafsson H Deng M Mills-Koonce WR Cox M. Bidirectional associations among sensitive parenting, language development, and social competence. *Infant Child Dev.* (2012) 21:374–93. 10.1002/icd.1750 25126021 PMC4128493

[43] Baydar N Akcinar B. Reciprocal relations between the trajectories of mothers’ harsh discipline, responsiveness and aggression in early childhood. *J Abnorm Child Psychol.* (2018) 46:83–97. 10.1007/s10802-017-0280-y 28215022

[44] Choi JK Kelley MS Wang D. Neighborhood characteristics, maternal parenting, and health and development of children from socioeconomically disadvantaged families. *Am J Commun Psychol.* (2018) 62:476–91. 10.1002/ajcp.12276 30239989

[45] Dawson-McClure S Calzada E Huang KY Kamboukos D Rhule D Kolawole B A population-level approach to promoting healthy child development and school success in low-income, urban neighborhoods: impact on parenting and child conduct problems. *Prev Sci.* (2015) 16:279–90. 10.1007/s11121-014-0473-3 24590412 PMC4156570

[46] Morrison J Pikhart H Ruiz M Goldblatt P. Systematic review of parenting interventions in European countries aiming to reduce social inequalities in children’s health and development. *BMC Public Health.* (2014) 14:1040. 10.1186/1471-2458-14-1040 25287010 PMC4203958

