# Supplementary material for: Responsive Caregiving and Opportunities for Early Learning Associated With Infant Development: Results From a Prospective Birth Cohort in China
Source: Front Pediatr. 2022 Jun 23;10:857107. doi: 10.3389/fped.2022.857107 (PMC9260074; doi:10.3389/fped.2022.857107)
Supplement: Supplementary file 1 [file Data_Sheet_1.pdf]

Supplementary Material

1 Supplementary Data

Mplus code (take the cross-lagged model of responsive caregiving and gross motor development for example)

```
 DATA:
    FILE IS MCPCDATA.dat;
 VARIABLE:
    MISSING ARE ALL (-99);
    NAMES ARE GMOTOR1 GMOTOR2 GMOTOR3 RESCARE1 RESCARE2 RESCARE3
    BBSEX BBDM BBGA BBW MOMAGE MOMEDU AFI;
    USEVAR ARE GMOTOR1 GMOTOR2 GMOTOR3 RESCARE1 RESCARE2 RESCARE3
    BBSEX BBDM BBGA BBW MOMAGE MOMEDU AFI;
  ANALYSIS:
    ESTIMATOR=MLR;
  MODEL:
    RESCARE2 ON RESCARE1;   !Responsive caregiving at wave 1->Responsive caregiving at wave 2
    RESCARE3 ON RESCARE2;   !Responsive caregiving at wave 2->Responsive caregiving at wave 3
    GMOTOR2 ON GMOTOR1;  !Gross motor at wave 1->Gross motor at wave 2
    GMOTOR3 ON GMOTOR2;  !Gross motor at wave 2->Gross motor at wave 3

    RESCARE2 ON GMOTOR1;   !Gross motor at wave 1->Responsive caregiving at wave 2
    RESCARE3 ON GMOTOR2;   !Gross motor at wave 2->Responsive caregiving at wave 3
    GMOTOR2 ON RESCARE1;   !Responsive caregiving at wave 1->Gross motor at wave 2
    GMOTOR3 ON RESCARE2;   !Responsive caregiving at wave 2->Gross motor at wave 3

    RESCARE1 WITH GMOTOR1;
    RESCARE2 WITH GMOTOR2;
    RESCARE3 WITH GMOTOR3;

    GMOTOR1 GMOTOR2 GMOTOR3 ON BBSEX BBDM
    BBGA BBW MOMAGE MOMEDU AFI;

  OUTPUT:
    SAMPSTAT STDYX;
```

2 Supplementary Figures and Tables

2.1 Supplementary Figures

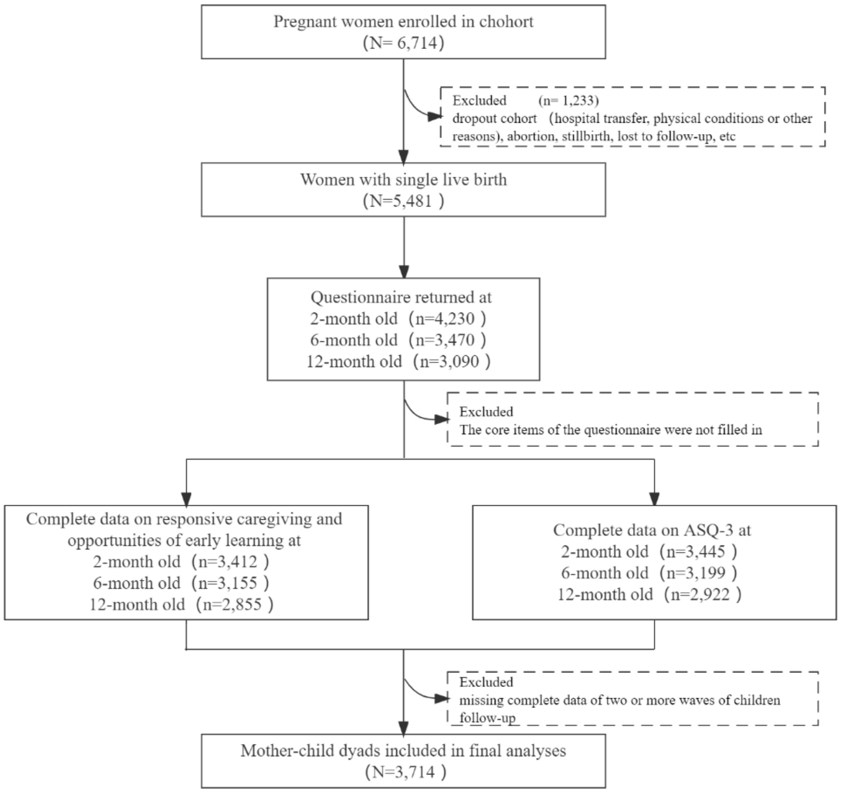

Supplementary Figure 1. Flow diagram for subject selection.

2.2 Supplementary Tables

Supplementary Table1. Demographic characteristics and repeated outcomes over three waves of follow-up

Variable	N (%)	P_{50} (P_{25}, P_{75})
Maternal age at delivery (years)		
⩽ 30	2586 (69.6)	
＞30	1128 (30.4)	
Maternal education level		
Middle school and below	274 (7.4)	
High school or same level	437 (11.8)	
Junior college or same level	1578 (42.5)	
College and above	1425 (38.3)	
Annual family income		
＜¥100,000	778 (20.9)	
¥100,000-200,000	1519 (40.9)	

¥200,000-300,000	1127 (30.4)	
⩾ ¥300,000	290 (7.8)	
Infant sex		
Male	1896 (51.1)	
Female	1818 (48.9)	
Delivery mode		
Natural delivery	1763 (47.5)	
Cesarean section	1951 (52.5)	
Gestational week (weeks)		
$<$ 37	157 (4.2)	
⩾ 37	3557 (95.8)	
Birthweight (g)		
$<$ 2500	97 (2.6)	
2500-4000	3370 (90.7)	
⩾ 4000	247 (6.7)	
At 2 months old		
Responsive caregiving		16 (13, 18)
Opportunities for early learning		14 (11, 18)
At 6 months old		
Responsive caregiving		4 (3, 5)
Opportunities for early learning		24 (21, 28)
At 12 months old		
Responsive caregiving		4 (4, 5)
Opportunities for early learning		28 (25, 32)
